# Transcendent Spirituality and Psychological Flexibility Among Adults on the Autism Spectrum in the United States

**DOI:** 10.1007/s10943-025-02448-7

**Published:** 2025-09-22

**Authors:** Heather H. Kelley, Ty B. Aller

**Affiliations:** Institute for Disability Research, Policy & Practice, Utah State University, Logan, USA

**Keywords:** Autism, Psychological flexibility, Spirituality, Transcendent spirituality, Satisfaction with life

## Abstract

Using structural equation modeling to analyze two waves of longitudinal data from 306 adults on the autism spectrum in the USA, we assessed whether transcendent spirituality (feeling connected to a higher being) was associated with psychological flexibility (the ability to respond to uncomfortable internal sensations in a flexible, values-driven manner), psychological inflexibility, and satisfaction with life over a 10-week period. While we identified no significant associations between transcendent spirituality and psychological inflexibility, we found that spirituality was longitudinally associated with psychological flexibility and satisfaction with life. Psychological flexibility mediated the association between spirituality and satisfaction with life. Implications are offered.

## Introduction

Adults on the autism spectrum are more likely to experience mental health concerns (MHC) when compared to adults who are not on the autism spectrum (Lai et al., 2019). Because of this, finding community supports and psychological interventions that help improve quality of life (QoL) while experiencing mental health concerns are a pressing issue for the autism community (Camm-Crosbie et al., 2019). Acceptance and Commitment Therapy (ACT), a strengths-based intervention that can improve QoL, has preliminary acceptability and efficacy for decreasing mental health concerns and improving QoL for adults on the autism spectrum (Byrne & O’Mahony, 2020). ACT is a therapy centered around increasing psychological flexibility (i.e., being able to accept and respond to internal stimuli including thoughts and feelings in an adaptable, functional, and values-driven manner; Hayes et al., 2012).

There are several salient similarities between spirituality and psychological flexibility, both in the underlying principles of the two constructs (e.g., finding a sense of meaning in pain) and the practices taught to increase both spirituality and psychological flexibility (e.g., meditation and mindfulness practices). Amidst numerous, systematic barriers to quality mental healthcare for adults on the autism spectrum (Adams & Young, 2020; Maddox et al., 2020), spirituality may be one way to promote psychological flexibility outside of the therapy room. However, limited research has explored whether spirituality is associated with psychological flexibility, and no research to our knowledge has explored spirituality and psychological flexibility among adults on the autism spectrum. As such, the purpose of the present study is to ascertain whether spirituality is associated with psychological (in)flexibility and satisfaction with life over time among adults on the autism spectrum using a two-wave longitudinal sample.

## Literature Review

### Adults on the Autism Spectrum

An estimated 5.5 million adults in the USA have been diagnosed with autism spectrum disorder (ASD; Centers of Disease Control & Prevention, 2022). ASD typically impacts how an individual interacts and communicates with other people, how they learn, and can influence a wide array of behaviors (e.g., ASD is often associated with repetitive movements). ASD can often lead to difficulties or discomfort in various environments, including work, school, and social functions (National Institute of Mental Health, 2023). There is an ongoing debate regarding whether to use identity-first (i.e., autistic adults) or person first (i.e., adults on the autism spectrum) language. As there is a lack of consensus on this language (Vivanti, 2020), we use adults on the autism spectrum, which research has found to be the most acceptable term to be used by someone not from the community (Bury et al., 2020).

Having an ASD diagnosis is associated with an increased likelihood of experiencing mental health concerns (Lai et al., 2019). However, many adults on the autism spectrum encounter numerous barriers to receiving treatment for MHC (Adams & Young, 2020; Maddox et al., 2020) and experience a high level of societal stigma when trying to connect with community resources (Frost et al., 2019). Given these barriers, there have been calls for more strengths-based research and supports for adults on the autism spectrum (Urbanowicz et al., 2019). In particular, given the challenges to accessing traditional healthcare and in-person mental health services (Adams & Young, 2020; Maddox et al., 2020; Whittle et al., 2019), research is needed to identify supports for mental health and QoL that are readily available outside of traditional healthcare settings. Given conceptual connections between spirituality and psychological flexibility, as described in the following sections, research is warranted that explores whether spirituality may be a meaningful resource for adults on the autism spectrum with MHC.

### Psychological Flexibility Among Adults on the Autism Spectrum

Societal constraints that are stigmatizing and harmful to the autism community may be a salient contributor to the increased prevalence of MHC among adults on the autism spectrum (Buck et al., 2014). While there are a number of therapeutic approaches that have been found to be effective for adults on the autism spectrum experiencing MHC, a common criticism of many of these approaches is their focus on changing the individual to meet the standards of a nonautistic society, rather than empowering adults on the autism spectrum with tools to help the individual integrate their unique thoughts and emotions into a fulfilling, values-driven life (Camm-Crosbie et al., 2019; Maddox et al., 2020).

Current research suggests that ACT is a promising approach at addressing this concern. Not only has research suggested that ACT is effective among adults on the autism spectrum experiencing MHC (Byrne & O’Mahony, 2020), but it is grounded in a strengths-based approach, which emphasizes psychological flexibility. By focusing on what matters most to an individual (their values), ACT-based interventions can help decrease psychological inflexibility (e.g., rigid thought patterns that divert an individual from living in line with their values) and help increase psychological flexibility (Hayes et al., 2012). This in turn can help improve the mental health and overall quality of life of adults on the autism spectrum (Aller et al., 2024). That said, in light of the barriers to traditional mental health services for adults on the autism spectrum described above (Adams & Young, 2020; Maddox et al., 2020; Whittle et al., 2019), there is a need to expand access to practices that can increase psychological flexibility beyond the therapy room. Research is needed to determine whether spirituality and spiritual connection may be one such practice.

### Spirituality in Adults on the Autism Spectrum

In understanding spirituality among adults on the autism spectrum, it is important to first distinguish secular spirituality from transcendent spirituality or religious spirituality. Most of the research related to spirituality among adults on the autism spectrum has investigated specific *secular* spiritual practices (e.g., mindfulness, meditation, yoga, etc.). Indeed, two systematic reviews have been conducted on secular spiritual practices such as mindfulness (Cachia et al., 2016) and yoga, meditation, and mindfulness-based interventions (Semple, 2019) for individuals on the autism spectrum. Across both reviews, benefits for reducing MHC (e.g., anxiety, depression) and improving social, emotional, or behavioral outcomes were identified. Less research has been conducted on religious or transcendent spirituality among adults on the autism spectrum. We identified only one study which compared secular spirituality to religious needs and practices. This study of 201 adults on the autism spectrum found that while secular spiritual practices (e.g., mindfulness exercises, meditation) played an important role in many of the participants’ lives, religious needs and practices were less common (Cwik & Büssing, 2019).

In reviewing the research on transcendent or religious forms of spirituality, it is important to first acknowledge that religious systems have been historically created and organized by nonautistic individuals and often lack inclusive policies, sensory experiences, and opportunities for neurodivergent adherents; recent work has highlighted several ways religious organizations can be more inclusive, with the primary suggestion being to better listen to autistic voices (Burnett, 2021; Unwin & Van Ommen, 2024).

Research has demonstrated how adults on the autism spectrum may approach religious beliefs and practices in unique ways, however, the limited research in this area presents inconsistent findings, making it difficult to draw clear conclusion regarding differences between in religiosity between individuals on the autism spectrum and those who are not on the autism spectrum (Burnos & Kopacz, 2025). A case study of four young adult men who identified as believers and had passed two non-confessional religious studies courses found that participants had a “stereoscopical” view, in which they joined two differing perspectives (i.e., spirituality or religion and natural sciences) to create their own model (Visuri, 2012). Participants also reported engaging in prayer but specified that they viewed prayer as a one-way line of communication (i.e., they speak to God and do not expect a response) and that prayer was a psychological or self-soothing tool; they expressed little interest in other religious rituals or practices.

Another study used survey data to investigate the image of God (i.e., the personal meaning the individual has ascribed to God) among 78 Dutch adults on the autism spectrum who had received treatment at a Christian mental health facility (Schaap-Jonker et al., 2013). They found that while adults on the autism spectrum reported more positive feelings toward God than negative feelings (i.e., anger and anxiety), they were more likely to report negative feelings toward God when compared to a nonclinical, nonautistic group. However, those who reported higher levels of religious salience were more likely to report positive feelings toward God. Taken together, the literature suggests that while adults on the autism spectrum may be less likely to identify as spiritual or find meaning in specific spiritual beliefs, spirituality remains important for a number of adults on the autism spectrum (Cwik, 2021). Given the limited research on this topic, it is important to further our understanding of the role transcendent spirituality and spiritual connection play in the well-being of spiritually oriented adults on the autism spectrum.

### Psychological Flexibility, Mental Health, and Spirituality

Connections between psychological flexibility and spirituality are an area in which research remains sparse. Our review of the literature identified primarily conceptual articles that highlight the similarities between Acceptance and Commitment Therapy (ACT), of which psychological flexibility is the theorized mechanism of action and spirituality. Arguably, the two most notable similarities between ACT and spirituality are their emphasis on value-based living and on finding meaning in pain (Kaplaner, 2019; Yavuz, 2022). Specifically, ACT consistently and intentionally helps individuals focus on staying present while ‘turning toward’ personally chosen values that encourages action toward more values-based ends. It recognizes that religious and spiritual beliefs and practices are an important source of values for many individuals and will often strive to connect to these values in helping individuals live in a values-based approach, so long as the individual is allowed to freely choose these characteristics (rather than having them be prescribed rules).

Regarding pain, ACT teaches that *pain* is an unavoidable human phenomenon and encourages individuals to acknowledge their pain and move toward accepting it. By being open, present, and accepting of this pain, individuals are able to begin to find meaning in their pain while continuing to create patterns of action that lead to valued ends (Nieuwsma et al., 2015). Psychological flexibility is viewed as a process of working with pain in a values-driven way that often promotes well-being. Transcendent spirituality, in which mundane or difficult experiences can be viewed through a lens which adds a significant meaning or purpose to these experiences as the individual feels connected to a higher being or power, similarly helps people work through pain. This meaning allows individuals to accept pain as an important part of their life. Indeed, many spiritual or religious individuals may express sincere gratitude for the pain they have experienced because of the meaning they have found in their pain (Kaplaner, 2019). Despite the similarities between psychological flexibility and spirituality, little research has investigated these constructs together, and to our knowledge, no research has investigated connections between spirituality and psychological flexibility among adults on the autism spectrum.

## Current Study

Given the lack of previous research on spirituality and psychological flexibility among adults on the autism spectrum, we first note the exploratory nature of this study, and highlight that our hypotheses are grounded largely in the conceptual connections that have been presented between spirituality and psychological flexibility. Using two waves of longitudinal data from a sample of adults on the autism spectrum, we investigated the following research questions:

- *RQ1*: What is the association between spirituality and psychological inflexibility and psychological flexibility among adults on the autism spectrum?
- *H1a*: We hypothesize that spirituality at T1 will be positively associated with psychological flexibility at T2.
- *H1b*: We hypothesize that spirituality at T1 will be negatively associated with psychological inflexibility at T2.
- *RQ2***:** What is the association between spirituality and satisfaction with life among adults on the autism spectrum?
- *H2*: We hypothesize that spirituality at T1 will be positively associated with satisfaction with life at T2.
- *RQ3*: Does psychological flexibility or psychological inflexibility mediate the association between spirituality and satisfaction with life?
- *H3*: We hypothesize the association between spiritualty and satisfaction with life will be mediated by psychological flexibility and psychological inflexibility.

## Method

### Procedures

This study utilizes two waves of data from a longitudinal study recruited in partnership by the Simons Foundation Powering Autism Research for Knowledge (SPARK) group, funded by the Simons Foundation Autism Research Initiative (SFARI). All procedures and questionnaires for this study were co-developed with self-advocates and approved by Utah State University’s Institutional Review Board (IRB# 11537) and the SFARI Participant Access Committee, though the research questions explored here are secondary questions and were not the focus of the initial proposal. The survey was hosted via SFARI’s online survey mechanism. On average, the survey took 25–30 min to complete. Recruitment materials described the study as voluntary, stated that 75 participants would be randomly selected to receive a $20 Amazon e-gift card, and was titled “Psychological wellness in adults with autism spectrum disorder during COVID-19.”

### Participants

To participate, participants were required to be at least 18 years of age, have an ASD diagnosis confirmed by SFARI, and be their own legal guardian. A total of 453 participants completed the online survey from September to November 2021. Ten weeks later, 306 participants completed the online survey for a second time (61.4% retention rate). Demographic questions were only asked at T1. Participant demographic characteristics are presented in Table 1.

**Table 1:** Participant Demographic Characteristics (N = 306)

Demographic Characteristics	n (%)
*Gender*	
Man	84 (28%)
Woman	162 (53%)
Gender Nonconforming	57 (19%)
*Relationship Status*	
In a romantic relationship	150 (49%)
Single	156 (51%)
*Sexual Orientation*	
LGBTQIA +	147 (50%)
Straight	146 (50%)
*Age*	
18–24	95 (31%)
25–29	71 (23%)
30–39	94 (31%)
40 +	46 (15%)
*Education*	
High school or less	51 (17%)
Trade or vocational school	10 (3%)
Some college or associate degree	126 (42%)
Four-year degree	65 (21%)
Graduate/professional degree	51 (17%)
*Income*	
Less than $20,000	(96) 31%
$21,000–65,000	124 (41%)
More than $65,000	86 (28%)
*Race/Ethnicity*	
White	283 (92%)
Hispanic or Latino	25 (8%)
Black	13 (4%)
Native American	16 (5%)
Asian	11 (4%)
Other	20 (7%)

As individuals could select more than one race or ethnicity, the total for race/ethnicity adds up to more than 100%. To use this variable in analyses, it was recoded as White (0) and Other (1). A number of participants selected both White and other racial or ethnic identities; in these situations, the participant was coded as other (1), resulting in 79% of the sample identifying as White, 21% as other

### Measures

#### Transcendent Spirituality

The survey used a four-item measure from the World Health Organization Quality of Life Spirituality, Religiousness, and Personal Beliefs (WHOQOL-SRPB) questionnaire (WHOQOL-SRPB Working Group, 2006). The base of these questions was, “To what extent does any connection to a spiritual being...” Following this base, the four items asked, “Help you get through hard times?”, “Help you to tolerate stress?”, “Help you to understand others?”, and “Provide you with comfort/reassurance?” Participants responded to these questions on a 5-point scale ranging from “not at all” to “an extreme amount.” We note the similarities of these measures to Fisher’s (2010) dimension of transcendent spirituality, and thus, we have termed these set of questions as transcendent spirituality, though for brevity, we often simply refer to it as ‘spirituality’ throughout the manuscript. McDonald’s omega was ω = .947 at T1.

#### Psychological (In)flexibility

We assessed both components of psychological flexibility and inflexibility to determine how these two might operate similarly or differently. Participants responded to 10 items from the Valuing Questionnaire (VQ; Smout et al., 2014) on a 7-point Likert scale (0 = Not at all True, 7 = Completely true). Half of the items from the scale make a *Values Progress* subscale (higher scores = higher psychological *flexibility*) and the other half make the *Values Obstruction* subscale (higher scores = higher psychological *inflexibility).* A sample item from the Values Progress subscale states, “I worked toward my goals even if I didn’t feel motivated to” while a sample item from the VQ Obstruction subscale states, “I spent a lot of time thinking about the past or future, rather than being engaged in activities that mattered to me.” McDonald’s omega was ω = .865 at T2 for the Values Progress subscale (psychological flexibility) and ω = .838 at T2 for the Values Obstruction subscale (psychological inflexibility).

#### Satisfaction with Life

The Satisfaction with Life Scale (Diener et al., 1985) was used to assess participants’ general satisfaction with their life. Participants responded to five-items on a 7-point Likert scale (1 = Strongly disagree, 7 = Strongly agree). Items include statements such as, “I am completely satisfied with my life” and “The conditions of my life are excellent.” McDonald’s omega was ω = .896 at T2.

### Analytic Approach

Descriptive statistics (means and standard deviations) and bivariate correlations were first obtained from the data (see Table 2). We note that despite our longitudinal data, we chose not to control for T1 of our endogenous variables. This decision was made based on our desire to understand associations over time rather than change overtime and based on the high correlation (> .80 for satisfaction with life, > .70 for psychological flexibility and psychological inflexibility) between the T1 and T2 measures which could mask significant associations (VanderWeele et al., 2020).

**Table 2:** Bivariate Correlations, Means, and Standard Deviations (N = 306)

Variables	(1)	(2)	(3)	(4)	(5)	(6)	(7)	(8)	(9)	(10)
1. Spirituality–T1	1.000									
2. Psych Flex—T2	.321^***^	1.000								
3. Psych Inflex—T2	−.076	−.449^***^	1.000							
4. SLS—T2	.238^***^	.677^***^	−.410^***^	1.000						
5. Race/ethnty (Other)	.005	.033	−.037	.020	1.000					
6. Relationship status (In)	−.020	.021	.024	.145^*^	−.062	1.000				
7. Income	−.221^***^	−.063	−.061	.083	−.032	.083	1.000			
8. Sex. Orient (LGBTQIA +)	−.259^***^	−.132*	.103	−.229^***^	−.002	−.003	−.021	1.000		
9. Education	−.141^*^	.007	−.091	−.002	−.030	.095	.236^***^	.027	1.000	
10. Age	−.024	.000	−.026	.009	.006	.315^***^	.071	−.119^*^	.255^**^	1.000
Mean	2.19	4.40	4.25	3.53	0.21	0.49	3.40	0.50	2.17	29.8
Standard Deviation	1.24	1.37	1.36	1.51	0.41	0.50	2.52	0.50	1.25	7.33

***p < .001. **p < .01. *p < .05

Next, we addressed our research questions through structural equation modeling (SEM). Specifically, we ran several models to assess model fit and address each of our research questions. First, we modeled confirmatory factor analyses (CFA) to assess factor loadings for our latent variables (spirituality, psychological flexibility, psychological inflexibility, and satisfaction with life) and to assess model fit. We set .40 as the cutoff for acceptable factor loadings (Hair et al., 2010) and evaluated the model fit based on standard criteria of CFI > .90 and a RMSEA < .08 (Little, 2013). We then ran a structural model investigating the association between spirituality and psychological flexibility (RQ1), after which we ran a structural model investigating the association between spirituality and satisfaction with life (RQ2). We then proceeded to add psychological flexibility as a mediator between spirituality and satisfaction with life, by regressing spirituality onto psychological flexibility and psychological flexibility onto satisfaction with life. To calculate indirect effects in the mediation analysis, 5,000 Monte Carlo replications and 95% confidence intervals were used, as recommended for robust mediational analyses in SEM (Kyriazos, 2018).

To address missing data, only those who completed both waves of the survey were retained for this analysis (N = 306). Individual item missingness was treated with full information maximum likelihood (FIML). Across the models, basic demographic information was controlled for (i.e., relationship status, sexual orientation, race, education, income, and age). All analyses were conducted in Stata 17.0 (Statacorp, 2022).

## Results

### Descriptive Statistics

Bivariate correlations, means, and standard deviations for each of our primary variables of interest as well as control variables are presented in Table 2. We found significant associations in the directions hypothesized for each of the primary variables of interest (i.e., spirituality, psychological flexibility, psychological inflexibility, and satisfaction with life).

### Structural Equation Models

Across we the four latent variables modeled, all factor loadings (see Table 3) were above .500 and thus deemed acceptable. We conducted a measurement model to assess model fit and found that the data fit the model well (χ^2^ (146) = 254.17, *p* < .001; CFI = .971; TLI = .966; RMSEA = .049, 90% CI: .039-.059). We proceeded to test our structural models.

**Table 3:** Latent Variable Factor Loadings

Items	
Spirituality–T1 *To what extent does any connection to a spiritual being…*	
Help you get through hard times?	0.942
Help you to tolerate stress?	0.92
Help you to understand others?	0.784
Provide you with comfort/reassurance?	0.941
Psychological Flexibility (*Values Progress*)–T2 *Over the past week…*	
I worked toward my goals even if I didn't feel motivated to	0.533
I was proud about how I lived my life	0.793
I made progress in the areas of my life I care most about	0.839
I continued to get better at being the kind of person I want to be	0.789
I felt like I had a purpose in life	0.731
Psychological Inflexibility (*Values Obstruction*)–T2 *Over the past week…*	
I spent a lot of time thinking about the past or future.	0.664
I was basically on "auto-pilot" most of the time	0.702
Difficult thoughts or feelings got in the way of what I really wanted to do	0.704
When things didn’t go according to plan, I gave up easily	0.622
It seemed like I was just 'going through the motions'.	0.822
Satisfaction with Life–T2	
In most ways, my life is close to my ideal	0.882
The conditions of my life are excellent	0.781
I am completely satisfied with my life	0.873
So far I have gotten the most important things I want in life	0.785
If I could live my life over, I would change nothing	0.601

Some items have been slightly modified for brevity

#### RQ1: Spirituality and Psychological (In)Flexibility

To address RQ1, we first regressed spirituality at T1 onto psychological flexibility at T2 and found that it had a significant, positive association (β = .31, *p* < .001), supporting H1a. None of the control variables were significantly associated with psychological flexibility. We also regressed spirituality at T1 onto psychological inflexibility at T2 and found no significant association between the two variables (β = -.11, *p* = .138), thus our results did not support H1b. None of the control variables were significant.

#### RQ2-RQ3: Spirituality and Satisfaction with Life

**Fig. 1:**
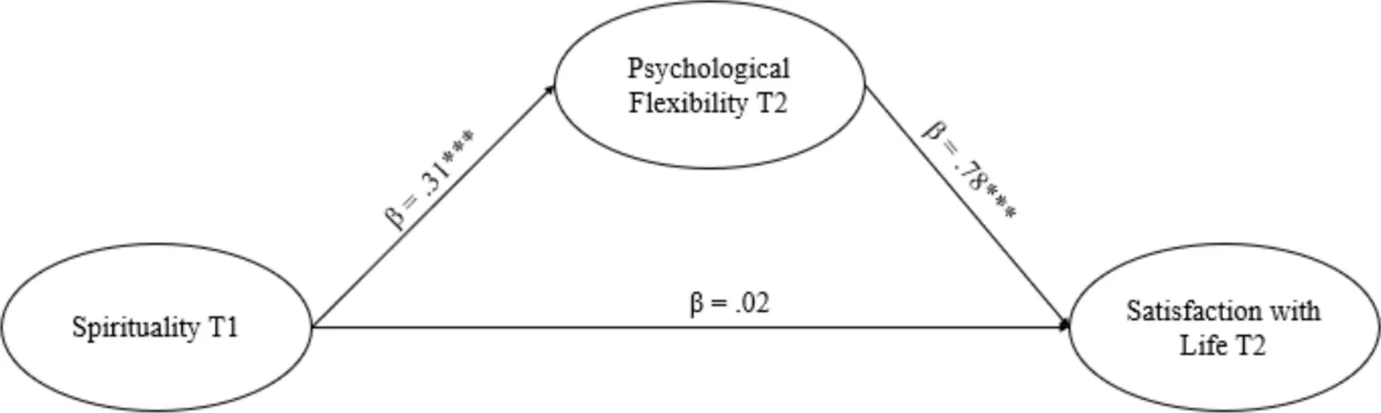
Mediation Model. *Note:* Not pictured for conciseness are the control variables (relationship status, sexual orientation, race, education, income, and age) which were correlated with Spirituality and regressed onto Psychological Flexibility and Satisfaction with Life

To address our second research question, we first assessed the impact of spirituality at T1 on satisfaction with life at T2. We found a significant, positive association between spirituality at T1 and satisfaction with life at T2 (β = .21, *p* = .001), supporting H2. Of the variables we controlled for, we found that being in a romantic relationship (β = .14, *p* = .002) and income (β = .13, *p* = .005) were positively related to satisfaction with life, while identifying as LGBTQIA + (β = −.12, *p* = .006) was negatively associated with satisfaction with life. We proceeded to test whether the association between spirituality and satisfaction with life was mediated by psychological flexibility (as the association between spirituality and psychological inflexibility was not significant, we did not test psychological inflexibility as a mediator). We found that psychological flexibility fully mediated (standardized indirect effect = .285, SE = .077, CI = .134:.436, *p* < .001) the association between spirituality and satisfaction with life as the direct effect was no longer significant (β = .018, *p* = .719; see Fig. 1), supporting H3.

## Discussion

We investigated the associations between transcendent spirituality, psychological flexibility, psychological inflexibility, and satisfaction with life among a sample of adults on the autism spectrum. While we found a significant association between spirituality at T1 and psychological flexibility at T2, there was not a significant association between spirituality and psychological inflexibility over time. Despite measures of psychological inflexibility often being reversed coded and labeled as ‘psychological flexibility’ in prior research (Cherry et al., 2021), this finding provides support for the argument that while related, psychological flexibility and psychological inflexibility are distinct constructs and should be treated as such. In line with conceptual arguments (Kaplaner, 2019), our findings also suggest that, for spiritually minded adults on the autism spectrum, focusing on their spiritual connections may be instrumental in helping them remain focused on living in accordance with what they value most during difficult times, such as when they are experiencing high levels of stress or an MHC. However, it appears that spirituality is less likely to help individuals in addressing rigid thought patterns, and thus, it is important that adults on the autism spectrum receive additional supports focused on working through inflexible thoughts that may be preventing them from acting in a values-driven manner.

We also found a significant association between spirituality and satisfaction with life over time. This association was fully mediated by psychological flexibility, suggesting that spirituality impacts satisfaction with life through increasing psychological flexibility, or more specifically, values progress, an important component of psychological flexibility (Hayes, 2012). Continued research should explore connections between psychological flexibility, spirituality, and satisfaction with life among adults on the autism spectrum.

Across the models, we included several salient demographic characteristics (i.e., relationship status, sexual orientation, race, education, income, and age) as control variables. Surprisingly, none of these controls were significantly associated with either psychological flexibility or psychological inflexibility. This may suggest that, among adults on the autism spectrum in the USA, the connections between spirituality, psychological flexibility, and psychological inflexibility do not systematically vary across SES and personal characteristics. Alternatively, such results may be obscured by the lack of diversity in our sample. It also suggests that there may be salient factors that were not accounted for in our models. We note that the data we used did not include any questions on religious affiliation or religiosity. Given the measure of spirituality used and its potential overlap with some religious beliefs, controlling for such measures may have produced insightful findings (Bertelli et al., 2020). When looking at satisfaction with life, we found that being in a romantic relationship and income were positively related to satisfaction with life, while identifying as LGBTQIA + was negatively associated with satisfaction with life. These results suggest that additional supports may be needed for single, lower income, and LGBTQIA + adults on the autism spectrum.

## Implications for Research and Practice

Liu et al.’s (2014) qualitative interviews with young people with I/DD and autism found that spiritual beliefs helped some of these individuals find meaning and value in their diagnosis. In conjunction with findings from the current study, this may suggest that clinicians working with spiritually minded adults on the autism spectrum who are struggling with MHC may find that honing into their clients’ spiritual beliefs and connections, along with the values behind these beliefs, could help their client better accept and find meaning in the challenges they are facing, including in their MHC. Perhaps more importantly in light of numerous barriers to care for MCH for adults on the autism spectrum (Adams & Young, 2020), spiritual connection may be a useful resource in helping individuals find this acceptance outside of the therapy room. Continued research on this is needed.

There are consistent calls to action for more strengths-based supports for adults on the autism spectrum. This study provides support for spirituality as one tool that, for spiritually minded individuals, may be helpful in increasing psychological flexibility and thus subsequently increasing overall satisfaction with life for adults on the autism spectrum. Given the exploratory nature of this study, continued research on how spirituality might be used in conjunction with other tools to improve psychological flexibility and satisfaction with life is needed.

## Limitations and Directions for Future Research

Despite some of the strengths of this study including the longitudinal design, there were several notable limitations. First, a salient limitation was the measure of spirituality used. We looked specifically at one dimension of spirituality that focused on connection with a spiritual *being*. This language may lend to specific religious connotations about God or a higher being and thus may preclude other secular dimensions of spirituality. Future research should include multidimensional measures of spirituality that span and distinguish secular and religious approaches to spirituality.

Second, while acknowledging the value in being able to look at the connections between our primary variables of interest over time, we note that there was not strong theoretical justification for the 10-week time gap. Future research should include additional waves of data and be intentional about the length of time between measurements. Relatedly, we note that this survey took place during the later part of 2021 when many COVID-19 restrictions were still in place in certain parts of the USA; it is unknown how these restrictions or general fear related to the pandemic may have influenced the results of this study. Finally, we note that we did not control for T1 of our endogenous variables and thus we could only look at the association over time rather than assessing change overtime.

We again note that exploratory nature of this study; while it provides preliminary, empirical support for theorized connections between psychological flexibility, spirituality, and satisfaction with life, more research on these constructs is needed. In particular, we are unable to assess the mechanism through which spirituality and psychological flexibility influence each other. Such research will be valuable in expanding our knowledge of how spirituality and psychological flexibility can be used together to help adults on the autism spectrum with MHC.

## Conclusion

The current literature suggests that while adults on the autism spectrum may be less likely to identify as spiritual than their counterparts who are not on the autism spectrum, spirituality remains a salient resource for many adults on the autism spectrum. While continued research is needed in this area, including with more robust, multidimensional measures of spirituality, our research suggests that for spiritually minded individuals, spiritual connection and beliefs may be useful resources that can help increase their psychological flexibility and satisfaction with life.
